# A perspective on terra incognita: uncovering the neuroanatomy of the human subcortex

**DOI:** 10.3389/fnana.2013.00040

**Published:** 2013-12-03

**Authors:** Anneke Alkemade, Max C. Keuken, Birte U. Forstmann

**Affiliations:** ^1^Department of Psychology, Cognitive Science Center Amsterdam, University of AmsterdamAmsterdam, Netherlands; ^2^Department of Neurophysics, Max Planck Institute for Human Cognitive and Brain SciencesLeipzig, Germany

**Keywords:** Human brain atlas, subcortical, *ex vivo*, *in vivo*

## Abstract

Recent exciting advancements in the field of *in vivo* neuroimaging allow for visualization of the living human brain with unprecedented anatomical detail. Large consortium studies will provide us with novel insights in the function and connectivity of the human brain. However, it is unlikely that the spatial resolution obtained using *in vivo* imaging will, in the near future, approximate the level of detail obtained in post-mortem anatomical studies. Initiatives such as the recently published Big Brain project (Amunts et al., 2013) herald a novel approach in post-mortem brain research. We feel that linking data from histological observations with *in vivo* imaging studies will greatly advance our understanding of the functional neuroanatomy of the human brain.

It is likely that *in vivo* imaging techniques will develop further during the next decade, allowing visualization of the human brain with unprecedented detail. Although the technical developments in *in vivo* brain imaging are exciting, the anatomical detail that is obtained remains limited. Increasing field strength of magnetic resonance imaging (MRI) scanners will improve the spatial resolution even further. However, it is unlikely that *in vivo* MRI will yield a level of detail comparable to histological approaches for studying the brain. At present a standard anatomical scan, obtained using a 3 Tesla MRI system, will take about 6 min and has an isotropic resolution of 1 mm. Such resolution does not allow the identification of individual neurons or glia, which can be achieved using histological techniques (Duyn, 2012). We argue that combining of post-mortem research with modern imaging techniques is needed to advance the research field of neuroscience.

Using state-of-the-art ultra-high resolution MRI, we can now visualize a number of small subcortical areas such as the subthalamic nucleus (Schafer et al., 2012). Although this is impressive, comparing these scans to images of histological staining of the same area puts *in vivo* MRI images into perspective. The detail obtained using ultra-high resolution MRI does not begin to compare to that of histology. Histological techniques have their limitations as well, and are typically used to obtain information about tissues in a 2D plane. Only small parts of the brain are usually studied for practical reasons. It seems only logical to combine the best of both worlds, but this rarely happens.

An unusually detailed, and impressive study was recently published by Amunts et al. (2013). In this study, an entire human brain obtained at autopsy was fixed, and cut in a coronal plane. Cytoarchitectonic structures were visualized using silver staining, microscope sections were photographed, and then with modern computational techniques further processed ultimately resulting in a 3D reconstruction with unprecedented detail, known as the Big Brain. This work is impressive not only in view of the spatial resolution it provides, but also in its laborious nature. More concretely, the resolution was 20 μm isotropic, required 1000 h of work, and resulted in 1 Tb of raw data. One can easily calculate the needed amount of work for the generalization of these results to the population. In this perspective article, we argue that such efforts are urgently needed to help define *terra incognita*, i.e., the human subcortex, in modern *maps* of the brain.

The lack of MRI research on small brain structures, especially subcortical structures, is reflected by their absence from brain atlases currently available for MRI research (Evans et al., 2012). We have illustrated this (Figure 1) by comparing an inventory of the subcortical gray matter structures depicted in standard MRI-atlases with the structures defined in the Federative Community on Anatomical Terminology (FCAT, 1998); a standardized nomenclature on human brain structures. The discrepancy should serve as a wakeup call for neuroscientists. We calculated that approximately seven percent of the structures mentioned in the FCAT are depicted in available standard MRI-atlases. This implies that these structures cannot be studied using automated analytical protocols available for MRI, and indicates the necessity of trained anatomists for the study of subcortical brain areas.

**Figure 1:**
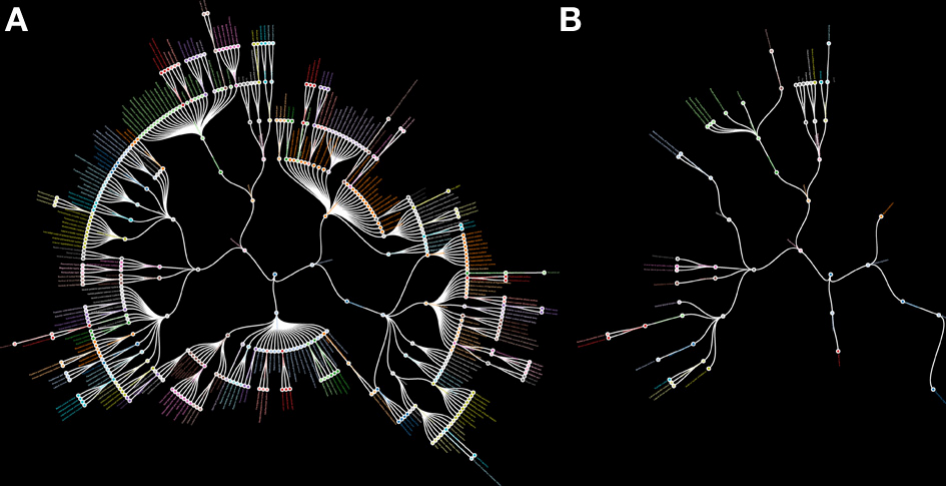
**Radial hierarchical tree of the human subcortex. (A)** A radial hierarchical tree illustrating the 455 subcortical structures as defined by the Federative Community on Anatomical Terminology (FCAT). The outer edges of the hierarchical tree display the individual subcortical gray matter nuclei. **(B)** A radial hierarchical tree illustrating seven percent of all subcortical structures as defined by the FCAT that are implemented as standard atlas maps in the three major MRI analysis software packages: FSL, SPM, and Freesurfer.

Neuroscientists around the world collaborate in order to fill our neuroanatomical knowledge-gap. The human connectome project is a 5 years collaborative project between 36 scientists from 11 different institutions, aimed to clarify the connectivity within the human brain in a cohort of 1200 healthy adults, using 3 and 7 Tesla MRI, diffusion tensor imaging, resting state fMRI, task fMRI, MEG, EEG, behavioral testing, DNA-analyses and network modeling (www.humanconnectomeproject.org). These studies are aimed to provide insight in the connectivity between gray areas within the normal human brain. The human connectome project will undoubtedly provide us with exciting new insights in the functional connections within the human brain. Such an ambitious approach would entail manual segmentation of all subcortical gray matter structures of the human brain. Our and other research groups are taking humble steps to start the mapping of subcortical structures in *in vivo* MRI scans (Cho et al., 2010; Kwon et al., 2012; Lenglet et al., 2012; Keuken et al., 2013). Our recent efforts indicate that mapping a single structure in 30 MRI scans takes approximately 4 months of work by two researchers. If we extrapolate these experiences to the human connectome project, it would take approximately 12,000 years of work to segment the 455 subcortical gray matter structures listed in the FCAT.

We live in an exciting era, and we feel that the continuing developments in functional and structural MRI research are invaluable for increasing understanding of the human brain. However, technical advancements in *in vivo* imaging do not abolish the need for classical, laborious anatomical studies. We believe that linking data obtained from the Big Brain project, the human connectome, in combination with other initiatives, such as the Allen Brain Atlas (www.brain-map.org), Blue Brain (bluebrain.epfl.ch), Human Brain project (www.humanbrainproject.eu), and the BRAIN initiative (www.nih.gov/science/brain) will contribute to improve our understanding of the human subcortex. Further developments in ultra-high field MRI stand at the brink of our journey of accurate and detailed mapping of the human brain. Obtaining detailed neuroanatomical maps of the human subcortex and registration of these maps into standard space will further facilitate and advance fMRI research.

## Conflict of interest statement

The authors declare that the research was conducted in the absence of any commercial or financial relationships that could be construed as a potential conflict of interest.
